# Antiproliferative Effects of Cynara Cardunculus in Colorectal Cancer Cells Are Modulated by the Circadian Clock

**DOI:** 10.3390/ijms23169130

**Published:** 2022-08-15

**Authors:** Luise Fuhr, Alireza Basti, Teresa Silva Brás, Maria F. Duarte, Angela Relógio

**Affiliations:** 1 Charité—Universitätsmedizin Berlin, Corporate Member of Freie Universität Berlin and Humboldt—Universität zu Berlin, Institute for Theoretical Biology, 10115 Berlin, Germany; 2 Charité—Universitätsmedizin Berlin, Corporate Member of Freie Universität Berlin and Humboldt—Universität zu Berlin, Medical Department of Hematology, Oncology, and Tumor Immunology, Molecular Cancer Research Center, 13353 Berlin, Germany; 3 Institute for Systems Medicine, Faculty of Human Medicine, MSH Medical School Hamburg—University of Applied Sciences and Medical University, 20457 Hamburg, Germany; 4 Alentejo Biotechnology Center for Agriculture and Agro-Food (CEBAL) and Instituto Politécnico de Beja (IPBeja), 7801-908 Beja, Portugal; 5 MED—Mediterranean Institute for Agriculture, Environment and Development & CHANGE—Global Change and Sustainability Institute, CEBAL, 7801-908 Beja, Portugal

**Keywords:** circadian clock, colorectal cancer, plant-derived compounds, proliferation, treatment sensitivity, apoptosis, cytotoxicity

## Abstract

The circadian clock generates 24 h rhythms in behavioural, cellular and molecular processes. Malfunctions of the clock are associated with enhanced susceptibility to cancer, worse treatment response and poor prognosis. Clock-controlled genes are involved in cellular processes associated with tumour development and progression including metabolism of drugs and the cell cycle. *Cynara cardunculus*, a plant of the *Asteraceae* family, has been reported to have antiproliferative effects on breast cancer cells. Here, we used the human colorectal cancer (CRC) cell line HCT116 and its knockout variants for different core-clock genes (*BMAL1*, *PER2*, *NR1D1*), to investigate the treatment effect of *C. cardunculus* lipophilic leaf extract under different clock scenarios. Our results show a direct effect of *C. cardunculus* on the circadian phenotype of the cells, as indicated by alterations in the phase, amplitude, and period length of core-clock gene oscillations. Furthermore, our data indicate a role for the circadian clock in sensitivity to *C. cardunculus* treatment. In particular, the treatment inhibited proliferation and induced cytotoxicity and apoptosis in a clock knockout-specific manner, in CRC cells. These results point to a potential effect of *C. cardunculus* lipophilic leaf extracts as a modulator of the circadian clock, in addition to its anti-proliferative properties.

## 1. Introduction

The circadian clock is an internal timing system that allows organisms to adapt physiology and behaviour to the geophysical time. The circadian timing system consists of a central pacemaker in the brain and peripheral clocks in every organ and cell. In mammals, a distinct set of genes is interconnected in regulatory feedback loops, thereby generating oscillations in gene expression in the core-clock itself, as well as in numerous target genes [1]. The clock influences many cellular processes including several hallmarks of cancer [2,3]. Among these are the cell cycle/cell proliferation and metabolism [4,5,6]. Malfunctions of the circadian clock are associated with different pathologies, including cancer and studies have linked the disruption of the clock to an enhanced susceptibility to develop cancer, bad treatment response and poor prognosis [7,8,9,10,11].

Metabolic reprogramming is strongly linked to tumour-specific signalling [12]. The ability of plant-derived natural compounds to modulate tumour cell metabolism and exert anticancer activity has been previously addressed using different cancer cell models [13,14,15]. Of all secondary metabolites found in plants, the sesquiterpene lactones (SL) group is one of the most prevalent and biologically significant, comprising over 5000 known compounds [16]. *Cynara cardunculus* L., commonly named cardoon, is a perennial plant, which belongs to the *Asteraceae* family, with vigorous growth and great adaptability to the Mediterranean climate. Cardoon is a recognized source of biologically active molecules, and previous work from our research team demonstrated, for the first time, that cultivated cardoon leaves are rich in sesquiterpene lactones (~94.5 g/kg dry weight), mainly represented by cynaropicrin [17]. Sesquiterpene lactones are one of the major groups of secondary metabolites found in plants, with recognized interest within pharmacology and medicine, due to a wide range of described biological activities, with different action mechanisms (synergistic and/or antagonistic affects), as well as molecular structure–activity relationships.

We have previously studied the antiproliferative effect of *Cynara cardunculus* extracts against triple negative breast cancer, demonstrating that cardoon leaf extracts had an inhibitory effect upon MDA-MB-231 cellular viability (IC50 10.39 µg/mL), also supported by the effect upon cell cycle phase distribution [18]. Cardoon leaf extracts led to a significant accumulation of MDA-MB-231 cells at G2 phase (59.5 ± 1.1%), representing a 4.7-fold increment in cell percentage compared with control. A detailed cell cycle analysis revealed a significantly higher expression of p21, p-Tyr15-CDK1 and cyclin B1 in MDA-MB-231 cells treated with cardoon leaf extracts, which ultimately was also associated with a p-Ser473-Akt expression decrease compared to control untreated cells [18].

Interestingly, previous studies from our research team reported non-cytotoxic effect of *Cynara cardunculus* leaf extracts when incubated with non-cancer human fibroblast (Bj5-ta) cell lines [19]. Other reports underscore the cancer-specific role of other sesquiterpene lactones in different cellular models. In particular, cynaropicrin has been demonstrated to be cytotoxic in a dose- and time-dependent manner in leukocyte cancer cell lines, while weakly blocking proliferation of human skin fibroblast (Detroit 55) cells [20]. Deoxyelephantopin, and isodeoxyelephantopin, two sesquiterpene lactones extracted from *Elephantopus scaber* L., were revealed not to be cytotoxic to normal human lymphocytes, only affecting the proliferating cells [21].

These promising insights suggest that extracts from cardoon leaves may be considered toward a natural-based therapeutic approach not only in triple negative breast cancer, but also in other cellular models.

Based on previous knowledge, we further aimed to address the role of *Cynara cardunculus* leaf extracts upon other cancer cell models. Colorectal cancer (CRC) is the third most common cancer, and the second leading cause of cancer death worldwide [22]. Several studies demonstrated the inter-connection between circadian rhythms and cancer, including CRC [8,9,23,24,25,26]. Taking into consideration the lack of knowledge regarding *Cynara cardunculus* leaf extracts and the circadian clock, in the present study we aimed to investigate if putative anti-cancer effects of *C. cardunculus* may be related to an underlying circadian regulation in CRC.

We used the CRC cell line HCT116 in a WT condition, as well as with knockouts (KOs) of different core-clock genes (*BMAL1*, *PER2*, *NR1D1*) to study the treatment effect of *C. cardunculus* lipophilic leaf extract on these cells. Our results point to a role for the circadian clock in sensitivity to *C. cardunculus* treatment.

Thus, our data suggest an anti-cancer effect of *C. cardunculus* on CRC cells, which seems to be clock-mediated in terms of treatment sensitivity. Interestingly, we also observe a bidirectional interplay between the clock and treatment, which has an effect on the clock phenotype.

## 2. Results

### 2.1. Treatment with C. cardunculus Leaf Extract Reduces Cell Proliferation and Induces Apoptosis and Cytotoxicity

**Figure 1:**
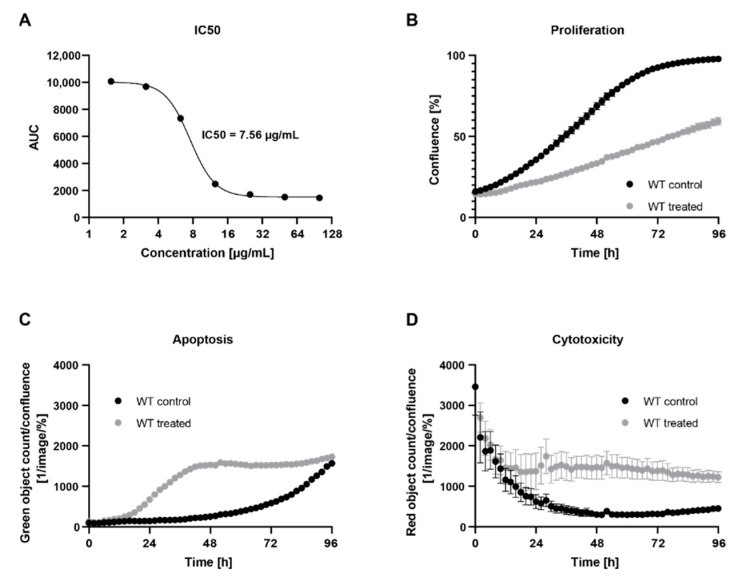
*C. cardunculus* treatment reduces cell proliferation and induces overall cell cytotoxicity and apoptosis in HCT116 cells. (**A**) IC50 determination of *C. cardunculus* treatment in HCT116-WT cells. Cells were treated with different concentrations of *C. cardunculus* lipophilic leaf extract and the IC50 value was calculated based on the AUC of proliferation curves determined using an Incucyte S3 Analyzer. (**B**) Proliferation, (**C**) apoptosis, and (**D**) cytotoxicity analyses after treatment with 7.5 µg/mL *C. cardunculus* lipophilic leaf extract in HCT116-WT cells over 4 days (*n* ≥ 6, mean ± SEM, *p* < 0.0001 for proliferation, apoptosis and cytotoxicity comparing AUC to control, one-way ANOVA with Tukey’s multiple comparisons test).

*Cynara cardunculus* lipophilic leaf extract has been shown to have anti-proliferative effects in human breast cancer cells lines [18]. We aimed to investigate the potential effects of *C. cardunculus* treatment in a human colorectal cancer cell model, namely in HCT116-WT cells. For this we measured the proliferation of cells when treated with *C. cardunculus* lipophilic leaf extract as compared to a vehicle control (Figure 1). A treatment concentration of 7.5 µg/mL was determined based on the IC50 value for the WT (Figure 1A) and led to an inhibition of cell proliferation. A comparison of the area under the curve (AUC) revealed a reduction of 45 % in WT cells after treatment (Figure 1B).

Subsequently, we evaluated whether the alterations observed in proliferation as a result of *C. cardunculus* treatment were due to induction of overall cell cytotoxicity and apoptosis as a specific type of cell death. For this purpose, we used two different live-cell imaging assays, using either a cytotox red reagent for counting dead cells and determine overall cell cytotoxicity or the Caspase-3/7 green apoptosis assay to measure cells undergoing caspase-3/7-mediated apoptosis. We measured apoptosis (Figure 1C) and cytotoxicity (Figure 1D) in cells treated with either 7.5 µg/mL *C. cardunculus* lipophilic leaf extract or with the vehicle control over 4 days. *C. cardunculus* treatment induced overall cell cytotoxicity and apoptosis in HCT116-WT cells, thus confirming the anticancer effect previously reported in human breast cancer cells [18].

### 2.2. Core-Clock Disruption Influences Response to C. cardunculus Treatment

Furthermore, recent publications from our group and others support the hypothesis that disruptions in the core-clock influence the sensitivity to anticancer treatment [8,27,28]. We thus tested this hypothesis with *C. cardunculus* leaf extract using a circadian model for HCT116 CRC cells, harbouring cells with different core-clock phenotypes. This was achieved by using core-clock knockout cells for three core-clock genes, namely HCT116-*BMAL1*-KO, HCT116-*PER2*-KO, and HCT116-*NR1D1*-KO.

**Figure 2:**
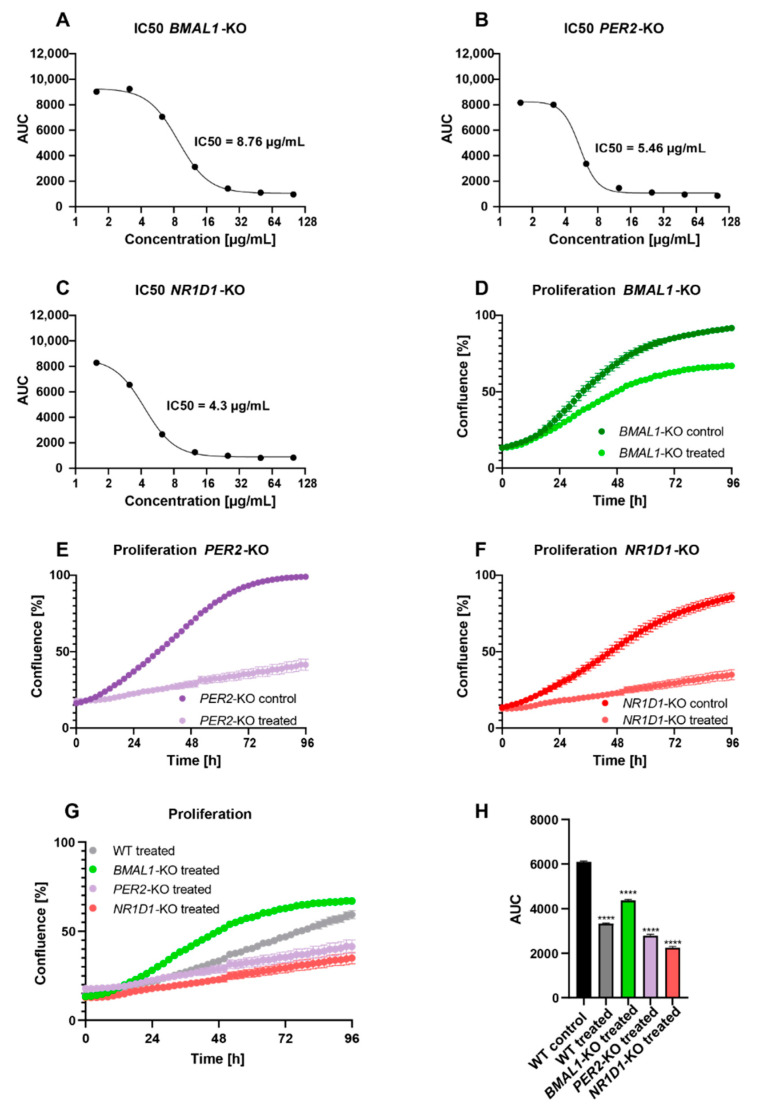
*C. cardunculus* treatment inhibits cell proliferation in HCT116 cells harbouring different core-clock KOs. IC50 determination of *C. cardunculus* treatment in (**A**) HCT116-*BMAL1*-KO cells, (**B**) HCT116-*PER2*-KO cells, and (**C**) HCT116-*NR1D1*-KO cells. Cells were treated with different concentrations of *C. cardunculus* lipophilic leaf extract and the IC50 value was calculated based on the AUC of proliferation curves determined using an Incucyte S3 Analyzer. Proliferation analysis of HCT116 cells after treatment with 7.5 µg/mL *C. cardunculus* lipophilic leave extract (**D**) in HCT116-*BMAL1*-KO cells, (**E**) in HCT116-*PER2*-KO cells, and (**F**) in HCT116-*NR1D1*-KO cells over 4 days. (**G**) Comparison of proliferation after treatment in WT and KO cells. (**H**) Barplot for proliferation comparing AUC between different conditions (*n* ≥ 6, mean ± SEM, **** *p* < 0.0001 for proliferation comparing AUC of treated WT and KOs to control WT, one-way ANOVA with Tukey’s multiple comparisons test).

In a first step, we determined the IC50 values for all KO conditions and observed a higher IC50 value for HCT116-*BMAL1*-KO (Figure 2A) cells as compared to HCT116-WT cells, while the IC50 values were lower for HCT116-*PER2*-KO (Figure 2B) and HCT116-*NR1D1*-KO (Figure 2C) cells.

The treatment led to an inhibition of proliferation. However, the effect of treatment varied between the different cell conditions and pointed to a core-clock gene-specific impact.

*C. cardunculus* treatment led to a reduction in proliferation of 24.5% in HCT116-*BMAL1*-KO cells (Figure 2D,G,H), 54.9% in HCT116-*PER2*-KO cells (Figure 2E,G,H), and 54.4% in HCT116-*NR1D1*-KO cells (Figure 2F–H), meaning that the knockouts of *PER2* and *NR1D1*, in particular, lead to a higher sensitivity to *C. cardunculus* treatment. These results are in line with the experimentally determined IC50 values for each cell condition.

**Figure 3:**
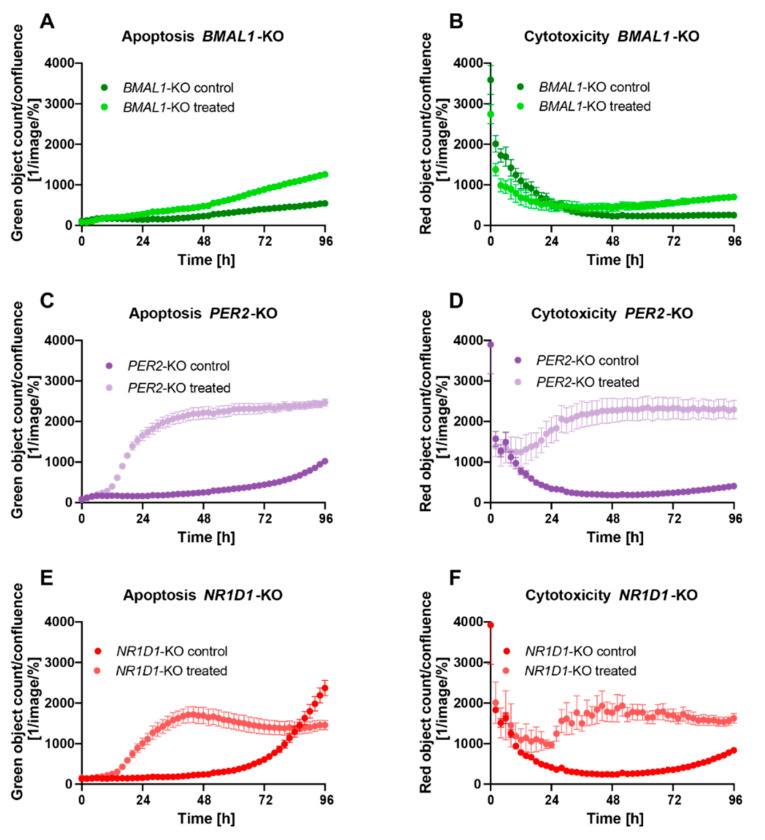
*C. cardunculus* treatment induces apoptosis and overall cell cytotoxicity in HCT116 cells. Apoptosis analysis of HCT116 cells after treatment with 7.5 µg/mL *C. cardunculus* lipophilic leaf extract in (**A**) HCT116-*BMAL1*-KO cells, (**C**) HCT116-*PER2*-KO cells, and (**E**) HCT116-*NR1D1*-KO cells. Measurements obtained by counting caspase3/7 green objects every 2 h over the course of 4 days using the IncuCyte. Cytotoxicity analysis of HCT116 cells after treatment with 7.5 µg/mL *C. cardunculus* lipophilic leave extract in (**B**) HCT116-*BMAL1*-KO cells, (**D**) HCT116-*PER2*-KO cells, and (**F**) HCT116-*NR1D1*-KO cells. Measurements obtained by counting red objects every 2 h over the course of 4 days using the IncuCyte (*n* ≥ 6, mean ± SEM).

In a next step, we evaluated the effect of *C. cardunculus* treatment on apoptosis and overall cell cytotoxicity in the different core-clock KO cells. Interestingly, the strength of the effect on apoptosis differed between the different KO conditions. While the treatment led to a significant induction of apoptosis in all conditions, the effect in HCT116-*PER2*-KO cells (Figure 3C) and HCT116-*NR1D1*-KO cells (Figure 3E) was stronger when compared to the effect of treatment on apoptosis in HCT116-*BMAL1*-KO cells (Figure 3A). As compared to treated WT cells, the effect of *C. cardunculus* treatment on apoptosis was higher in HCT116-*PER2*-KO cells, while the effect was very low in HCT116-*BMAL1*-KO cells (Figure S1A).

The effects of *C. cardunculus* treatment on overall cell cytotoxicity were consistent with the results obtained from the apoptosis assay (Figure 3B,D,F and Figure S1B), suggesting that overall cell cytotoxicity results from the activation of caspase-3/7-mediated apoptosis in this cell model system. The induction of cytotoxicity was particularly high in HCT116-*PER2*-KO cells, as compared to treated HCT116-WT cells. In contrast to this, very low levels of cytotoxicity were measured in HCT116-*BMAL1*-KO cells upon treatment.

### 2.3. C. cardunculus Alters Circadian Properties in HCT116 Cells

**Figure 4:**
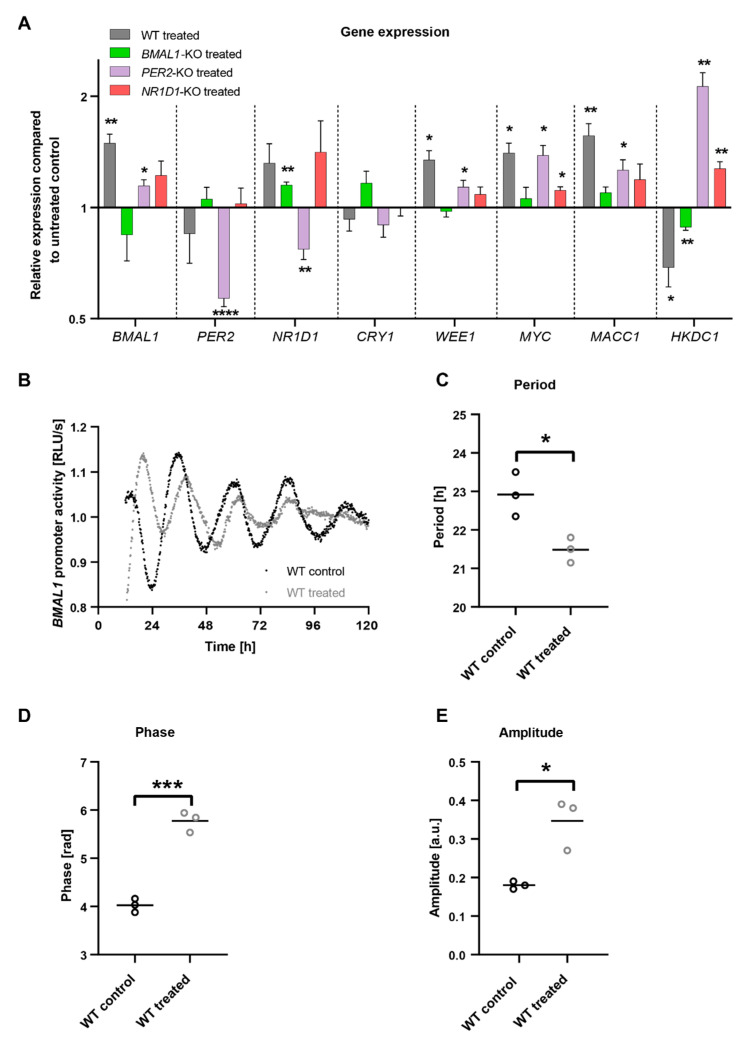
*C. cardunculus* treatment affects circadian gene expression and *BMAL1*-promoter activity in HCT116-WT cells. (**A**) Gene expression analysis of selected genes in HCT116-WT, HCT116-*BMAL1*-KO, HCT116-*PER2*-KO and HCT116-*NR1D1*-KO cells after treatment with *C. cardunculus* (*n* = 3, mean ± SEM). Data are shown compared to the corresponding untreated control cells. (**B**) Cells were lentivirally transduced with a *BMAL1*-luciferase construct (BLP) and synchronized with medium change. Cells were treated either with a vehicle control or with 7.5 µg/mL *C. cardunculus* lipophilic leaf extract. Bioluminescence was measured for five consecutive days. Displayed is one representative replicate for each condition. Scatter dot plot of (**C**) Period [h], (**D**) phase [rad] and (**E**) amplitude of circadian oscillations of *BMAL1*. * *p* < 0.05, ** *p* < 0.01, *** *p* < 0.001, **** *p* < 0.0001, two-tailed unpaired *t*-test. See also Figure S2.

In a next step, we investigated possible connections between elements of the circadian clock network and *C. cardunculus* treatment. To this end, we treated HCT116 cells and analysed the expression of different circadian core-clock genes (Figure 4A). *C. cardunculus* treatment induced changes in the expression of core-clock (*BMAL1*, *PER2*, *NR1D1* and *CRY1*), as well as cell cycle related genes (*WEE1* and *MYC*), the metastasis-related gene *MACC1* and the metabolic gene *HKDC1*. The induced effects differed between different KO conditions. Our results show that the treatment induces alterations in the expression of the core-clock genes in a KO-dependent manner. In particular, the treatment significantly induced *BMAL1* expression in WT cells (*p* < 0.01, *n* = 3, mean ± SEM), while only slightly affecting other core-clock genes. The KO of *PER2* led to significant expression changes in almost all core-clock genes analysed after treatment (*BMAL1*, *PER2* and *NR1D1*), while in *BMAL1* KO cells only *NR1D1* was significantly upregulated upon treatment (*p* < 0.01, *n* = 3, mean ± SEM). Interestingly, all other analysed genes were significantly affected by the treatment in WT cells. While *WEE1*, *MYC* and *MACC1* were upregulated, *HKDC1* was decreased after treatment. *HKDC1* was significantly altered in all KO conditions as well. While it was increased in *PER2* and *NR1D1* KO cells (*p* < 0.01, *n* = 3, mean ± SEM), it showed decreased expression in *BMAL1* KO cells after treatment (*p* < 0.01, *n* = 3, mean ± SEM). This pointed towards an effect of *C. cardunculus* treatment on the core-clock and cancer related genes, with a clock-KO-specific role in HCT116 cells.

To analyse the effects of *C. cardunculus* treatment on the circadian clock itself, we stably transduced HCT116 cells with a Luciferase-reporter under the control of the *BMAL1* promoter. Using this approach, the *BMAL1* promoter activity was measured in living cells and circadian parameters such as phase, period and amplitude were determined. Remarkably *C. cardunculus* treatment induced changes in *BMAL1*-promoter activity in HCT116-WT cells (Figure 4B). The treatment induced a significant decrease in period length, Figure 4C) and a significant phase delay (Figure 4D). However, the amplitude was higher upon treatment (Figure 4E).

**Figure 5:**
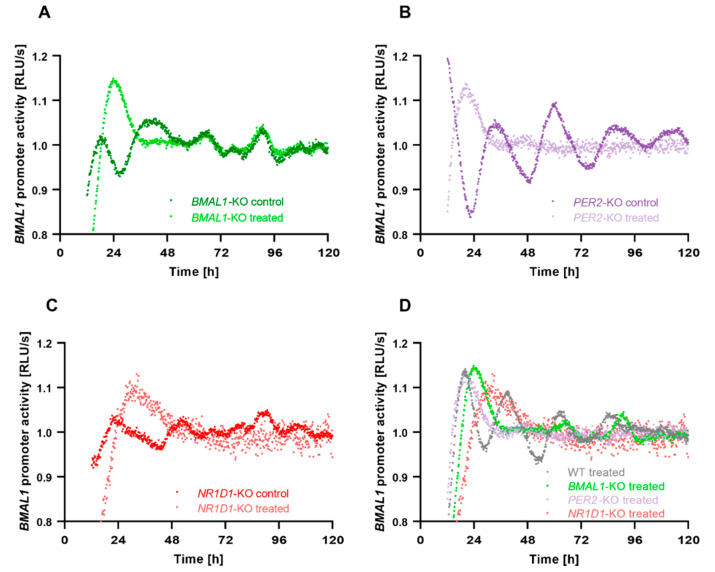
*C. cardunculus* treatment affects *BMAL1*-promoter activity in HCT116 cells harbouring different core-clock knockouts. Cells were lentivirally transduced with a *BMAL1*-luciferase construct (BLP) and synchronized with medium change. Cells were treated either with a vehicle control or with 7.5 µg/mL *C. cardunculus* lipophilic leaf extract. Bioluminescence was measured for five consecutive days. Displayed is one representative replicate for each condition. (**A**) Bioluminescence recordings of *BMAL1*-promoter activity in HCT116-*BMAL1*-KO cells, (**B**) bioluminescence recordings of *BMAL1*-promoter activity in HCT116-*PER2*-KO cells, (**C**) bioluminescence recordings of *BMAL1*-promoter activity in HCT116-*NR1D1*-KO cells, and (**D**) bioluminescence recordings of *BMAL1*-promoter activity in treated WT and KO cells. See also Figure S2.

To further analyse the effect of *C. cardunculus* treatment on circadian oscillations in HCT116 cells harbouring different core-clock knockouts, HCT116-*BMAL1*-KO, HCT116-*PER2*-KO and HCT116-*NR1D1*-KO cells were transduced with the *BMAL1*-LUC reporter construct and the promoter activity of *BMAL1* was measured over five consecutive days (Figure 5). In agreement with the results described above, all HCT116-KO cells showed changes in *BMAL1* promoter activity upon *C. cardunculus* treatment. Although the period and phase of the oscillations could not be determined in treated KO cells, the strong damping of oscillations in treated KO cells indicates a loss of synchrony and subsequent cell death.

## 3. Discussion

Cancer treatment remains a societal and health challenge and will gain even more importance in the coming years due to the increasing cancer incidence, according to the WHO’s International Agency for Research on Cancer (https://www.iarc.who.int/ accessed on 1 July 2022). As such, there is a need for new products and adjuvants in cancer treatment to increase efficacy and reduce side effects of cancer therapy.

The circadian system is an emerging field in cancer therapy, as it plays an important role in drug metabolism and consequently in treatment toxicity, tolerability and efficacy of treatment [7,11,29]. However, the role of natural plant extracts upon circadian clock modulation is far from being understood. A recent study used 137 crude drug extracts to identify circadian clock modulators using a human osteosarcoma U2OS cell line stably expressing the clock reporter *BMAL1*-dLuc [30], thus pointing to a putative involvement of plant extracts upon modulation of the circadian clock.

Here, we show that treatment with *C. cardunculus* induces a promising anti-proliferative effect, which needs to be further investigated in future in vivo studies. Namely, our results show an induction of cytotoxicity and apoptosis and inhibition of proliferation in HCT116-WT cells, and HCT116 cells harbouring different core-clock KOs. Interestingly, the effects observed were specific for the particular core-clock KO cells. In particular, *BMAL1*-KO cells seem to be less sensitive to *C. cardunculus* treatment, as seen by the effect on proliferation, apoptosis, cytotoxicity and a higher IC50 value, as compared to WT and other core-clock KO cells. These observations are in line with previous studies pointing to a role for *BMAL1* as a tumour suppressor and sensitivity to anticancer treatment [8,27,28]. In contrast to this, *PER2*-KO and *NR1D1*-KO cells were more sensitive to *C cardunculus* treatment, as indicated by their lower IC50 values. Our results suggest an anti-cancer effect of *C. cardunculus* in a CRC model, which is mediated by the circadian clock, as shown by different effects of treatment in different CRC core-clock KO cells. Moreover, the observed levels of caspase-3/7-mediated apoptosis and overall cell cytotoxicity were comparable, we therefore suggest that apoptosis is the major type of cell death being induced upon *C. cardunculus* treatment.

Remarkably, *C. cardunculus* has a direct effect on the circadian clock in these CRC cells, since treatment affected core-clock gene expression, as well as circadian oscillations. Especially in HCT116-WT cells, we observed a strong influence of *C. cardunculus* treatment on the circadian phenotype, as seen by significant changes in the period, phase and amplitude. Additionally, we observed changes in core-clock gene expression upon treatment, further pointing to a strong influence of *C. cardunculus* treatment on the core-clock machinery in these CRC cells.

Other genes involved in cancer proliferation and metastasis also showed alterations in their expression. HKDC1 is a member of the hexokinase family that plays a role in glucose metabolism [31]. Several studies postulate a role for HKDC1 as a potential therapeutic target for different cancers [32,33]. Previous results from our group point to a role for *HKDC1* as a mediator of circadian clock-induced metabolic phenotype rewiring in cancer cells and subsequent modulation of tumour progression and drug response [8]. Here, the expression of the hexokinase *HKDC1* was downregulated in WT and *BMAL1*-KO cells upon treatment, whereas it was upregulated in *PER2*-KO and *NR1D1*-KO cells. The higher IC50 values for WT and *BMAL1*-KO cells and lower IC50 values for *PER2*-KO and *NR1D1*-KO cells might be partly mediated by *HKDC1*, leading to a higher treatment sensitivity when upregulated upon treatment and to a lower sensitivity when downregulated upon treatment. It would thus be possible that the effect of *C. cardunculus* might be mediated through *HKDC1*. MACC1 is a known driver for cancer metastasis, especially in colon cancer, as well as other solid tumours [34,35], and is considered to be a therapeutic target in CRC restricting metastasis [36]. A differential *MACC1* expression in HCT116 cells upon *C. cardunculus* treatment, as seen in WT and *PER2*-KO conditions, points towards the effect of *C. cardunculus* on clock and metastasis-related components with likely effects on cancer progression.

As previously reported, *C. cardunculus* leaf lipophilic extracts are particularly rich in sesquiterpene lactones (SLs). The chemical structures of SLs allow for different target interactions, mainly due to the α-methylene-γ-lactone moiety, making SLs able to modulate different cellular responses. At the molecular level, SLs impact different signal pathways, being the NF-kB, MAPK, and JAT-STAT the most well-described ones [37]. The impact of *C. cardunculus* leaf extracts on the circadian clock had not been addressed in previous studies.

CRC cell lines display a variety of circadian phenotypes which range from a completely disrupted clock to less dramatic alteration in circadian rhythms, which have been reported in published work [8,9,23,24,25]. This variability can also be observed when evaluating the connection between the circadian clock and cancer-related pathways, and the effects are often specific for certain cell lines, as well as specific core-clock gene disruptions [8,38]. Although we focus on the HCT116 cell line and its derived core-clock KO cell lines in this study, future studies should include other CRC cell lines for possible generalizations of the treatment effect of *C. cardunculus* in CRC.

It is well accepted that the metabolic pathways specifically altered in tumours may represent vulnerabilities that can potentially be targeted with minimum damage to healthy cells [8,12,39,40]. The desired specificity towards cancer cells underscores the importance of developing tumour-selective drug delivery systems for these plant extracts, as successfully exploited for other plant-derived anticancer drugs [41].

The results of our study highlight *C. cardunculus* as a potential adjuvant for cancer therapy and reinforce the link between treatment effectiveness and the circadian timing system, underlining the need for individual cancer therapy based on the circadian phenotype of the patient. Future studies in other in vitro and in vivo cancer models will be needed to further investigate and potentially validate the effect of *C. cardunculus* as a therapeutic approach in cancer.

## 4. Materials and Methods

### 4.1. Cell Culture

HCT116 cells (ATCC^®^ CCL-247™) were cultured in Dulbecco’s Modified Eagle Medium DMEM (Gibco, Thermo Fisher Scientific, Waltham, MA, USA) supplemented with 10% FBS (Gibco, Thermo Fisher Scientific, Waltham, MA, USA) and 1% Penicillin−Streptomycin (Gibco, Thermo Fisher Scientific, Waltham, MA, USA) in a humidified atmosphere containing 5% CO_2_ at 37 °C. For live-cell bioluminescence recording, cells were maintained in phenol red-free DMEM (Gibco, Thermo Fisher Scientific, Waltham, MA, USA) containing 10% FBS, 1% Penicillin−Streptomycin and 250 µM D-Luciferin (Bio-Rad laboratories, Hercules, CA, USA). Cell counting and morphology analysis were performed in a LUNA™ Automated Cell Counter (Logos Biosystems, Anyang, South Korea).

HCT116 core-clock KO cell lines were generated as previously described [42,43]. In short, HCT116 WT cells were transfected with CRISPR-Cas9 plasmids and guide RNAs targeting core-clock genes *BMAL1*, *PER2* or *NR1D1*. Positive cells were single-cell sorted based on GFP expression, expanded and validated for KO efficiency on DNA, RNA and protein level.

### 4.2. Determination of Treatment Concentration

Treatment concentrations were determined based on the experimentally determined IC50 value. For the experimental determination of *C. cardunculus* treatment concentration, cells were seeded in 96-well plates one day prior to treatment (2500 cells/well). On the next day cells were synchronized by medium change and treated with 100 µg/mL, 50 µg/mL, 25 µg/mL, 12.5 µg/mL, 6.25 µg/mL, 3.13 µg/mL or 1.56 µg/mL *C. cardunculus* lipophilic leaf extract. Proliferation was measured over 6 days in an IncuCyte S3 analyser and the IC50 values were determined based on the AUC of each treatment condition. For subsequent experiments, a treatment concentration of 7.5 µg/mL was used. Control cells were treated with a vehicle control (Ethanol). For RNA extraction, cells were synchronized and treated for 48 h before RNA extraction. For all other experiments, cells were synchronized by medium change and treated directly before start of the experiment/measurement.

### 4.3. Lentivirus Production

Lentiviral elements containing a *BMAL1*-promoter-driven luciferase (BLP) were generated as previously described [44]. For lentivirus production, HEK293T (human, kidney, ATCC Number: CRL-11268) cells were seeded in 175 cm^2^ culture flasks and co-transfected with 12.5 μg packaging plasmid psPAX, 7.5 μg envelope plasmid pMD2G and 17.5 μg expression plasmid using the CalPhos mammalian transfection kit (Clontech, Mountain View, CA, USA) according to the manufacturer’s instruction. To harvest the lentiviral particles, the supernatant was centrifuged at 4100× *g* for 15 min to remove cell debris and passed through a 45 μm filter (Sarstedt, Nümbrecht, Germany). The lentiviral particles were stored at −80 °C.

### 4.4. Transduction with Lentiviral Vectors

For lentiviral transduction, 1 × 10^5^ cells were seeded in 6-well plates. On the day of transduction, 1.5 mL of supernatant of the corresponding lentivirus were added to each well. 8 μg/mL protamine sulfate (Sigma-Aldrich, St. Louis, MO, USA) and 4 μg/mL polybrene (Sigma-Aldrich, St. Louis, MO, USA) was used to enhance transduction efficiency. After 48 h, the medium was replaced, and selection medium was added (complete growth medium containing appropriate antibiotic) to obtain stably transduced cells and incubated at 37 °C with 5% CO_2_ atmosphere. Untransduced cells treated with the same antibiotic concentration were used as selection controls.

### 4.5. Bioluminescence Measurements

For live-cell bioluminescence recordings, 2.5 × 10^5^ HCT116 cells were seeded in 35 mm dishes and maintained in phenol red-free DMEM (Gibco, Thermo Fisher Scientific, Waltham, MA, USA) containing 10% FBS, 1% Penicillin-Streptomycin supplemented with 250 µM D-Luciferin (Bio-Rad laboratories, Hercules, CA, USA). Cells were synchronized by adding fresh medium prior to measurement and treated with *C. cardunculus* or a vehicle control where appropriate. *BMAL1*-promoter-(BLP)-reporter activities were measured, using a LumiCycle instrument (Actimetrics, Wilmette, IL, USA) for five consecutive days. Raw luminescence data were de-trended using the 24 h running average (divided values) using Chronostar analysis software V3.0 (Kramer lab, Berlin, Germany) [45]. The first 12 h of measurement were removed from the analysis, since the first data collection is comparatively very noisy due to technical limitations of the device. The phase in radian was calculated using the following equation with φ(h) = phase (in h), T = period:

φ(rad) = φ(h)⋅(2⋅πT)

### 4.6. RNA Extraction, cDNA Synthesis (Reverse Transcription) and Quantitative Real-Time PCR (qPCR)

For RNA extraction and gene expression analysis, 2 × 10^5^ cells per well were seeded in 12-well plates one day prior to treatment. On the next day, cells were synchronized by medium change and treated with *C. cardunculus* or vehicle control for 48 h.

Total RNA was isolated using the RNeasy Plus Mini kit (Qiagen, Hilden, Germany) according to the manufacturer’s manual. Prior to the purification procedure, medium was discarded, and cells were washed with PBS and lysed directly in RLT Plus buffer (Qiagen, Hilden, Germany) supplemented with 2-Mercaptoethanol (AppliChem, Darmstadt, Germany). Genomic DNA was digested using gDNA eliminator columns provided with the kit (Qiagen, Hilden, Germany). RNA was eluted in 30 µL RNase-free water. The final RNA concentration was measured using a Nanodrop 1000 (Thermo Fisher Scientific, Waltham, MA, USA). RNA was then stored at −80 °C until use. Then, 1 µg of total RNA was reverse-transcribed to cDNA with M-MLV reverse transcriptase (Invitrogen, Thermo Fisher Scientific, Carlsbad, CA, USA), random hexamers (Thermo Fisher Scientific, Waltham, MA, USA) and dNTPs Mix (Thermo Fisher Scientific, Waltham, MA, USA). RT-qPCR was performed using human QuantiTect Primer assays (Qiagen, Hilden, Germany) and SsoAdvanced Universal SYBR Green Supermix (Bio-Rad laboratories, Hercules, CA, USA) in 96-well plates. GAPDH was used as reference genes. The following primers were designed in-house:

*MACC1*: FW-TTCTTTTGATTCCTCCGGTGA, REV-ACTCTGATGGCA-TGTGCTG.

*WEE1*: FW- CCCCGCCACACAAGACCT, REV-GAGAGCAAACTCTTGGGCGTG.

The following QuantiTect Primer assays (Qiagen, Hilden, Germany) were used:

*GAPDH*: QT00079247; *BMAL1*: QT00011844; *PER2*: QT00011207; *NR1D1*: QT00000413; *CRY1*: QT00025067; *MYC*: QT00035406; *HKDC1*: QT00086359

The qPCR reaction and the subsequent melting curve were performed using a CFX Connect Real-Time PCR Detection System (Bio-Rad laboratories, Hercules, CA, USA). A melting curve analysis was performed to detect potential unspecific amplification products. Cq values were determined using the regression method. The expression levels were normalized to those of GAPDH (ΔCT) and calibrated in relation to the respective control (ΔΔCT). Relative quantification was calculated using the 2^−ΔΔCt^ method. Biological and technical replicates were included in the analysis. The mean and the standard error of the mean were calculated.

### 4.7. Proliferation, Apoptosis, and Cytotoxicity Measurements

For proliferation assays, 2500 cells/well (HCT116-WT, HCT116-*BMAL1*-KO, HCT116-*PER2*-KO, HCT116-*NR1D1*-KO) were seeded in a 96-well plate (Sarstedt, Nümbrecht, Germany). Cells were incubated overnight. On the next day cell medium was replaced by fresh medium containing appropriate treatment or control substances (*C. cardunculus* lipophilic leave extract or ethanol) and cells were placed in the IncuCyte^®^ S3 Live Cell System (Sartorius, Göttingen, Germany). Four images per well were recorded every two hours using the phase channel and 10× objective. Analysis was performed using the IncuCyte S3 Software V2019B (Sartorius, Göttingen, Germany).

For apoptosis assays, 2500 cells/well (HCT116-WT, HCT116-*BMAL1*-KO, HCT116-*PER2*-KO, HCT116-*NR1D1*-KO) were seeded in a 96-well plate (Sarstedt, Nümbrecht, Germany). Cells were incubated overnight. On the next day cell medium was replaced by fresh medium containing appropriate treatment or control substances (*C. cardunculus* lipophilic leaf extract or ethanol) as well as caspase 3/7 (Sartorius, Göttingen, Germany, 1:1000). Cells were placed in the IncuCyte^®^ S3 Live Cell Analysis System (Sartorius, Göttingen, Germany). Four images per well were recorded every two hours using the phase and green fluorescence channel and 10x objective. Analysis was performed using the IncuCyte S3 Software (Sartorius, Göttingen, Germany).

For cytotoxicity assays, 2500 cells/well (HCT116-WT, HCT116-*BMAL1*-KO, HCT116-*PER2*-KO, HCT116-*NR1D1*-KO) were seeded in a 96-well plate (Sarstedt, Nümbrecht, Germany). Cells were incubated overnight. On the next day, cell medium was replaced with fresh medium containing appropriate treatment or control substances (*C. cardunculus* lipophilic leave extract or ethanol) as well as cytotox red dye (Sartorius, Göttingen, Germany, final well concentration: 250 nM). Cells were placed in the IncuCyte^®^ S3 Live Cell System Analysis (Sartorius, Göttingen, Germany). Four images per well were recorded every two hours using the phase and red fluorescence channel and 10× objective. Analysis was performed using the IncuCyte S3 Software (Sartorius, Göttingen, Germany).

### 4.8. Cynara cardunculus Plant Extracts

*Cynara cardunculus* L. (DC) leaves were collected in March 2021 at an installed *Cynara cardunculus* field in Serpa, Portugal. Before extraction, samples were freeze-dried in a freeze drier (Scanvac coolsafe, Labogene, Denmark). Dried leaves were grounded using a domestic mixed grinder (Moulinex, France).

*Cynara cardunculus* leaf extracts were produced as described by Bras et al. [46]. Briefly, Ultrasound Assisted Extraction was performed at a solid/liquid ratio of 1/27 (g/mL), a duty cycle of 25%, an amplitude of 67% and extraction temperature of 44 °C. Extraction time was 30 min. Obtained extract was filtered and solvent evaporated at low pressure on a rotary evaporator (Hei-VAP Advantage, Heidolph, Germany).

### 4.9. Statistical Analysis

All experiments were carried out with at least three biological replicates for each condition. All results are represented as mean ± SEM. Statistical analysis of the results was performed using Graphpad Prism version 9 software (GraphPad Software, San Diego, CA, USA) using either one-way ANOVA followed by Tukey’s multiple comparisons test or by two-tailed unpaired *t*-test, based on the experimental design. A *p*-value < 0.05 was considered as statistically significant. (* = *p* < 0.05; ** = *p* < 0.01; *** = *p* < 0.001, **** = *p* < 0.0001). Cell proliferation, apoptosis and cytotoxicity was analyzed by comparing area under the curve (AUC) data between different conditions.

## Data Availability

Not applicable.

## Supplementary Materials

The following supporting information can be downloaded at: https://www.mdpi.com/article/10.3390/ijms23169130/s1.
