# Supplementary material for: Antiproliferative Effects of Cynara Cardunculus in Colorectal Cancer Cells Are Modulated by the Circadian Clock
Source: Int J Mol Sci. 2022 Aug 15;23(16):9130. doi: 10.3390/ijms23169130 (PMC9409071; doi:10.3390/ijms23169130)
Supplement: Supplementary file 1 [file ijms-23-09130-s001.zip › ijms-1832126-supplementary.pdf]

A

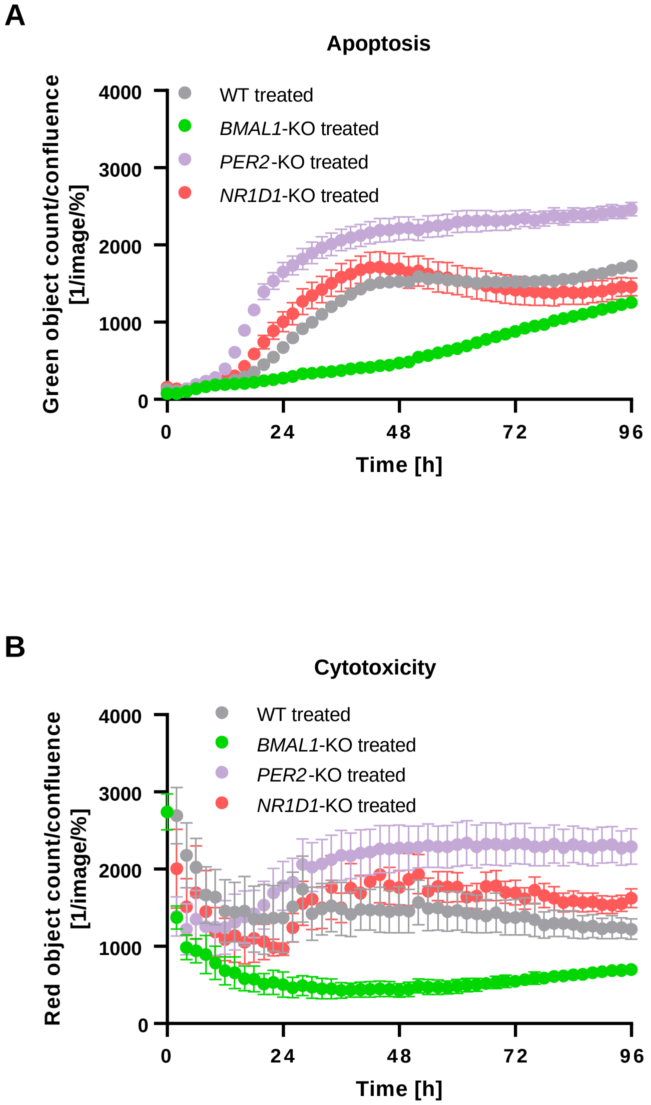

Figure S1. **Effect of *C. cardunculus* treatment on apoptosis and overall cell cytotoxicity.** (**A**) all treated conditions (WT and KOs) (n ≥ 6, mean ± SEM, p < 0.0001 for apoptosis comparing AUC of treated HCT116-*BMAL1*-KO cells and HCT116-*PER2*-KO cells to treated WT, one-way ANOVA with Tukey`s multiple comparisons test). (**B**) all treated conditions (WT and KOs) (n ≥ 6, mean ± SEM, p < 0.02 for cytotoxicity comparing AUC of treated Kos to treated WT, one-way ANOVA with Tukey`s multiple comparisons test).

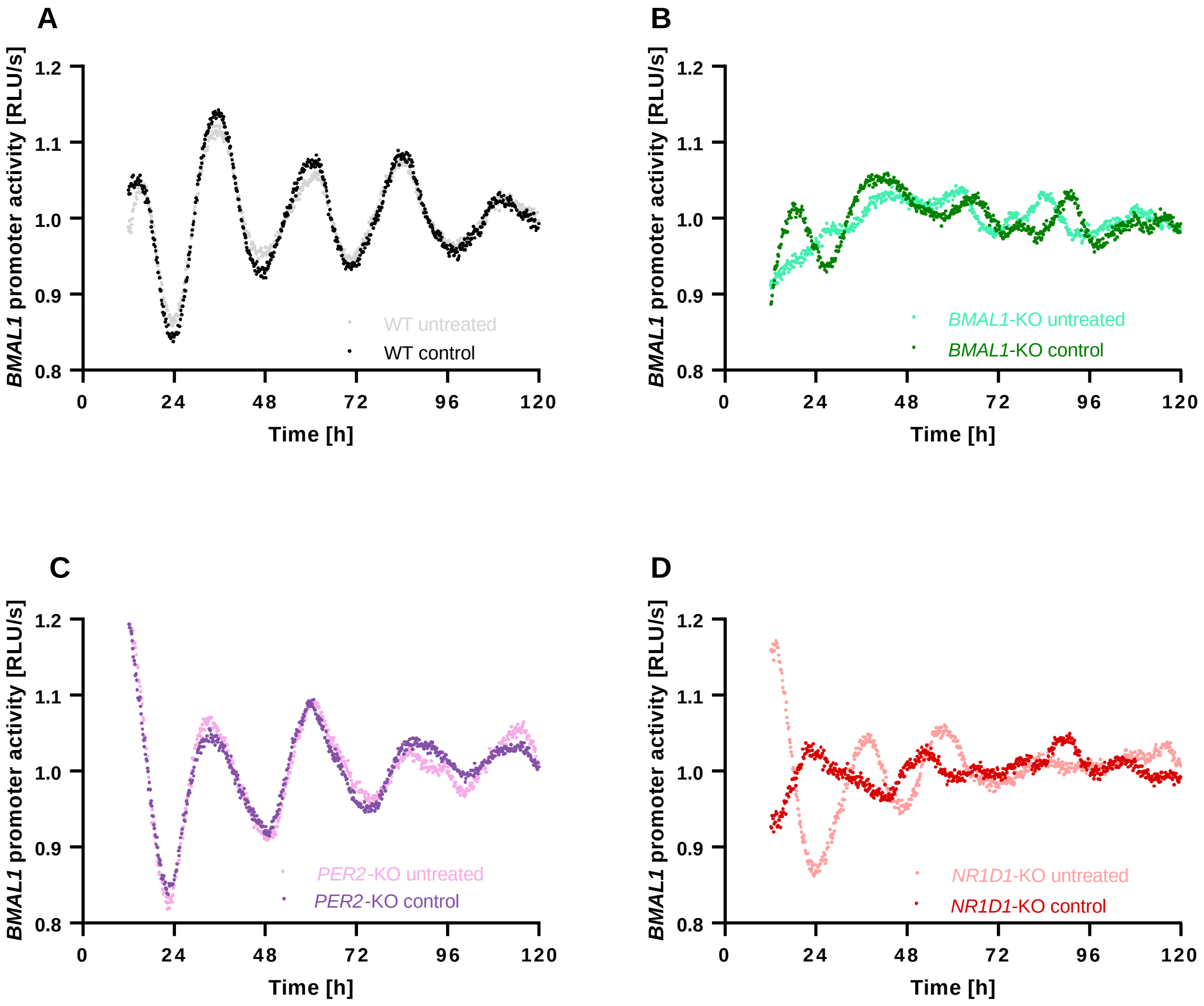

Figure S2. ***BMAL1*-promoter activity in HCT116 cells harboring different core-clock knockouts.** Cells were lentivirally transduced with a *BMAL1*-luciferase construct (BLP) and synchronized with medium change. Cells were either untreated or treated with a vehicle control (ethanol). Bioluminescence was measured for five consecutive days. Displayed is one representative replicate for each condition. (**A**) Bioluminescence recordings of *BMAL1*-promoter activity in HCT116-WT cells, (**B**) Bioluminescence recordings of *BMAL1*-promoter activity in HCT116-*BMAL1*-KO cells, (**C**) Bioluminescence recordings of *BMAL1*-promoter activity in HCT116-*PER2*-KO cells, (**D**) Bioluminescence recordings of *BMAL1*-promoter activity in HCT116-*NR1D1*-KO cells.
